# The Perils of Ablative Stereotactic Radiosurgery for the Nucleus Accumbens: A Historical Perspective Urging Caution

**DOI:** 10.7759/cureus.98236

**Published:** 2025-12-01

**Authors:** Bomin Sun, Bart Nuttin, Hemmings Wu, Antonio De Salles, Michael Schulder, John Adler

**Affiliations:** 1 Neurosurgery, Center for Functional Neurosurgery, Ruijin Hospital, Shanghai Jiao Tong University School of Medicine, Shanghai, CHN; 2 Neurosurgery, University Hospitals Leuven, Leuven, BEL; 3 Neurosurgery, ZAP Surgical Systems, San Carlos, USA; 4 Neurosurgery, University of California, Los Angeles, Los Angeles, USA; 5 Neurosurgery, Donald and Barbara Zucker School of Medicine at Hofstra/Northwell, Hempstead, USA; 6 Radiation Oncology, ZAP Surgical Systems, San Carlos, USA

**Keywords:** accumbens, drug addiction, obsessive-compulsive disorders, psychiatric disease, stereotactic and functional neurosurgery, stereotactic radiosurgery (srs)

## Abstract

Over the past decade, interest in lesion-based neuromodulation has experienced a resurgence fueled in large part by the noninvasive nature of new technologies, especially high-intensity focused ultrasound (HIFU). The noninvasiveness and relatively low cost of these outpatient methods are key attributes contributing to growing acceptance. A recent resurgent embrace of stereotactic radiosurgery (SRS) for brain lesioning, especially in the treatment of tremor via thalamotomy, builds on this new trend. While we believe this development for treating well-established movement (tremor) and behavioral (obsessive-compulsive) disorders is warranted and perhaps even applauded, we are worried that enthusiasm for lesioning using SRS may now be getting ahead of itself. This concern stems from the exuberance for using SRS to treat additional behavioral diseases, as showcased during the recent American Society for Radiation Oncology (ASTRO) 2025 meeting. The near giddiness observed in San Francisco regarding the use of SRS to treat patients with psychiatric disorders prompts us to write the present cautionary editorial. The aim of this editorial is to caution against premature clinical enthusiasm for using ablative SRS to target the nucleus accumbens (NAc) for psychiatric disorders. Our objectives are to (1) contextualize this proposal within the troubling history of psychosurgical overreach, (2) summarize the substantial neuroscientific and clinical risks inherent to NAc ablation, and (3) advocate for a strict, evidence-based, and ethically grounded approach that prioritizes reversible neuromodulation over irreversible lesioning.

## Editorial

Interest in lesion-based neuromodulation has surged over the past decade, propelled by the arrival of genuinely noninvasive tools like high-intensity focused ultrasound (HIFU). The noninvasiveness and relatively low cost of these outpatient approaches have accelerated their clinical adoption. In parallel, stereotactic radiosurgery (SRS) has seen renewed enthusiasm for lesioning applications, particularly for thalamotomy in the treatment of tremor, reflecting this broader trend. Ablative SRS, a procedure that uses highly focused beams of radiation to precisely target and destroy a small, specific area within the brain, has rightly earned its place as a powerful tool in modern functional neurosurgery, offering a minimally invasive solution for selected severe, pharmacologically refractory neurological disorders [1]. Ventral intermediate nucleus (VIM) thalamotomy for essential tremor, trigeminal rhizotomy for trigeminal neuralgia, and anterior capsulotomy for intractable obsessive-compulsive disorder (OCD) are good examples of radiosurgery's transformative potential. It is this legacy of success that appears to have encouraged the recent and deeply unsettling proposal at the 2025 American Society for Radiation Oncology (ASTRO) Annual Meeting to explore ablative SRS targeting of the nucleus accumbens (NAc) for patients with selected psychiatric indications such as OCD and addiction.

The NAc, undeniably a critical node in the brain's reward circuitry, presents an alluring target for treating conditions characterized by severe motivational deficits, anhedonia, or addiction. However, the apparent enthusiasm for this newest iteration on functional ablation appears to have arisen without any awareness of the history of 20th-century psychosurgery. While two generations ago many severely afflicted patients worldwide were helped by frontal lobotomy, without question, many were also badly injured by the zeal of neurosurgeons, neurologists, and psychiatrists seeking in desperation effective treatment for major psychiatric conditions [2]. The ensuing generational regulatory and popular backlash against ALL psychiatric neurosurgery has meant that despite persistent neurosurgical interest, only a limited number of research procedures, done under rigid institutional review board (IRB) approval, have been performed in recent decades. Without proper knowledge and safeguards, therapeutic radiation risks traveling a near-identical ruinous frontal-lobotomy path for patients, who notably, by nature of their disorders of mood and behavior, tend to lack conventional standards of self-agency. Ultimately, if we fail to learn from history, we could now be imperiling the emerging field of stereotactic radiation and its potential to benefit countless suffering individuals.

The NAc, a core component of the ventral striatum, is not a simple "on/off" switch for addiction and disorders of mood; rather, it is the primary integration hub for essential processes that define human motivation, learning, and emotional processing. It acts as the final common pathway where highly salient inputs, dopamine from the ventral tegmental area (VTA) conveying reward prediction error, glutamate from the prefrontal cortex (PFC) governing cognitive control, and inputs from the amygdala processing emotion, converge. Its primary cells, GABAergic medium spiny neurons (MSNs), are critical for transforming these converging signals into goal-directed behaviors [3]. An ablated NAc does not merely stop pathological urges; it fundamentally cripples the capacity for essential, "human" functions, including forming positive reinforcement-based memories, sustaining natural motivation for essential activities like work or relationships, and experiencing the full range of pleasure (hedonic capacity) [3]. Importantly, psychiatric disorders reflect dysfunction across distributed brain networks, as opposed to being attributable to a single, discrete "pathological" structure. Ultimately, any damage to the NAc risks rendering the patient an emotionally flat automaton, unable to access the very mechanisms of motivation and reward necessary for a meaningful life.

The most compelling argument against clinical trials involving NAc ablation lies not in theory, but in the devastating results already documented on a large scale. The early 2000s saw an alarming, unregulated surge in ablative neurosurgery for addiction across several centers in China, overwhelmingly targeting the NAc [4]. While initial reports from designated centers did claim short-term success in curbing dependence, providing a basis for subsequent enthusiasm, the long-term clinical trajectory was marked by severe and lasting iatrogenic injury [5,6]. Reports from this period indicated that several thousands of patients underwent radiofrequency ablation of this reward center in an aggressive, yet ultimately disastrous, attempt to curb drug dependence [7]. The consequences were so serious and widespread, compounded by the procedure's rapid, unregulated adoption by inexperienced surgeons and a failure in securing proper informed consent, that they prompted the highest levels of the Chinese federal government to issue a blanket ban on the procedure by 2004. The long-term side effects reported were not merely a therapeutic inconvenience; they often constituted a wholesale functional loss, including catastrophic personality changes, profound loss of motivation, emotional flattening, and anhedonia so severe that it constituted a permanent impairment to the patient's quality of life. The clinical evidence from this massive, albeit uncontrolled, experiment provides a clear and non-negotiable cautionary tale: ablating the NAc carries an unacceptably high risk of trading one severe pathology (addiction) for another (a permanent emotional and volitional deficit). To ignore this tragedy and embrace a new ablative approach to the NAc, albeit with fancy new technology, is to demonstrate a willful ignorance of fundamental neuroscience and indefensible clinical decision-making.

Given the irreversible nature of ablation, the scientific and regulatory communities have a profound ethical and clinical imperative to prioritize non-destructive neuromodulatory approaches to the NAc. However, during the above-mentioned ASTRO 2025 plenary session, it was implied during a lecture titled "Precision Without Incision: The New Era of Functional Radiosurgery" that lesion-based SRS could be a logical tool for soon treating behavioral disorders such as depression and addiction [8]. Despite the current global mental health and addiction crises, desperation does not justify permanent, high-risk interventions that lack a sound neurobiologic basis. If targeted radiation is to be explored at all for neuromodulation, at a minimum, it must be based on sound preclinical science and then under the most rigorous mechanisms of human research oversight. More to the point, we advocate for the study of "non-ablative" approaches, which, at the same time, MUST include medical specialists with deep knowledge of both the complex neuroscience and the clinical ramifications of all forms of neuromodulation [9-12]. To paraphrase the creator of SRS, Lars Leksell, "A fool with even the best of tools is still a fool". Whether the NAc is ultimately the optimal target for treating selected behavioral disorders (as opposed to the anterior limb of the internal capsule and ventral striatum), any proposal seeking to modulate this structure (NAc) for psychiatric purposes must begin and end with modulatory objectives, as opposed to lesioning [13,14].

Despite the strong admonition this editorial is intended to embody, it is recognized that circumstances could change in the future. As knowledge of NAc anatomy and function evolves, and presumably with further improvements in lesioning technology, it might become possible for some exceptionally experienced functional neurosurgical center to reexamine the potential for lesioning well-defined discrete NAc subregions. However, even under these circumstances, the requisite clinical research must be conducted within the most stringent clinical research framework, especially as it pertains to ethical oversight. Given this final caveat, it bears repeating that all lesion-based interventions involving any part of the NAc, regardless of ablation technology, should be deemed an absolute last resort, justified only after reversible neuromodulatory options, which offer the critical advantages of fully adjustable and non-permanent effects, have been exhausted. This fundamental reversibility is the core ethical safeguard, ensuring that if a patient experiences catastrophic side effects, the intervention can be mitigated or halted, thus preserving their long-term quality of life and autonomy. Ablation, whether by radiofrequency or high-dose radiosurgery, violates one of the first principles of modern functional neurosurgery, which is to maintain the highest degree of modulatability to minimize the risk of permanent iatrogenic harm.

Future research should prioritize the development of highly focal, non-lesional neuromodulatory strategies aimed at NAc subregions. Furthermore, efforts must concentrate on establishing rigorous neuroimaging biomarkers that can objectively identify and predict the necessary modulation target within the distributed brain networks underlying disorders like addiction and OCD. Ultimately, multi-center trials focused on these highly controlled, reversible, and personalized non-ablative approaches represent the responsible direction for advancing functional neuromodulation in psychiatry.

In conclusion, the history of ablative neurosurgery for psychiatric disorders has been a long, fraught journey, marked by both cautious progress and catastrophic setbacks. While ablative SRS has proven effective in some carefully selected functional disorders, the proposal to target the NAc represents an attempt to pursue a failed procedure under the delusion of technological advancement. The documented outcomes from a now 20-year-old Chinese experience serve as a stark, undeniable record of clinical failure, a failure rooted in the fundamental, indispensable role the NAc plays in motivation, emotion, and cognition. This history means that, at least for now, the notion of making lesions in the NAc is both physiologically misguided and clinically hazardous. The pathway forward in psychiatric neuromodulation must be defined by prudence, precision, and, critically, modulatability. The ethical responsibility to protect patients from permanent, functional harm demands nothing less than this conservative and historically informed approach.
